# La thymoglobuline en traitement d’induction chez les transplantés rénaux à faible risque immunologique: une expérience marocaine

**DOI:** 10.11604/pamj.2022.41.138.23091

**Published:** 2022-02-17

**Authors:** Zineb Abouzid, Mohamed Anass Amar, Maher Abdessater, Meryem Alioubane, Anissa Benjaafar, Naima Ouzeddoun, Loubna Benamar, Rabia Bayahia, Tarik Bouattar

**Affiliations:** 1 Service de Néphrologie-Dialyse-Transplantation Rénale, Centre Hospitalier Universitaire Ibn Sina, Rabat, Maroc,; 2 Service d´Urologie, Centre Hospitalier René Dubos, Pontoise, France,; 3 Faculté de Médecine et de Pharmacie, Université Mohammed V, Rabat, Maroc

**Keywords:** Thymoglobuline, traitement d’induction, transplantation rénale, Thymoglobulin, induction treatment, renal transplantation

## Abstract

**Introduction:**

la thymoglobuline® est un anticorps polyclonal indiqué en traitement d´induction en transplantation rénale. Le but de notre travail est d´étudier l´efficacité de la thymoglobuline® en traitement d´induction chez des transplantés rénaux à faible risque immunologique.

**Méthodes:**

il s´agit d´une étude rétrospective entre Janvier 2012 et Septembre 2017. On a inclus les patients à faible risque immunologique, défini par l´absence de transplantation antérieure et d´anticorps spécifiques du donneur (DSA), qui ont reçu un traitement d´induction à base de thymoglobuline®. On a étudié les caractéristiques démographiques et cliniques, les paramètres biologiques ainsi que les complications post transplantation rénale.

**Résultats:**

nous avons inclus 55 patients transplantés rénaux avec un suivi moyen de 38 ± 16 mois. L´âge moyen était de 39,1 ± 12,1 ans avec une prédominance masculine (58,2%). Aucun patient n´avait de DSA avant la greffe. La dose cumulative de la thymoglobuline® était de 4,26 ± 0,87 mg/kg avec une durée moyenne de 5 ± 0,82 jours. La déplétion lymphocytaire était maximale au premier jour de la perfusion. Nous avons recensé 3 cas de reprise retardée de la fonction du greffon, au moins un épisode d´infection bactérienne chez 56,4% de nos patients, 7 cas d´infection à cytomégalovirus (CMV) (12,7%) et 2 cas de maladie à CMV (3,6%). La survie du greffon était notée chez tous nos patients avec un taux de créatinine sérique moyen à 11,7 ± 3,6 mg/l au cours de la dernière consultation.

**Conclusion:**

bien qu´elle ne soit pas indiquée en première intention chez les patients à faible risque immunologique, la thymoglobuline® peut néanmoins être prescrite à dose plus faible, avec une efficacité similaire et sans exposition à un risque de rejet plus élevé.

## Abstract (en)

**Introduction:**

Thymoglobulin® is a polyclonal antibody indicated for induction treatment in kidney transplantation. The purpose of this study is to estimate the effectiveness of Thymoglobulin® as induction treatment in kidney transplant patients with low immune risk.

**Methods:**

we conducted a retrospective study between January 2012 and September 2017. Patients with low immunological risk, defined as the absence of previous transplantation and donor-specific antibodies (DSA), were included and received Thymoglobulin® induction therapy. Demographic and clinical characteristics, biological parameters and post-renal transplant complications were studied.

**Results:**

we enrolled 55 kidney transplant patients with an average follow-up period of 38 ± 16 months. The average age of patients was 39,1 ± 12,1 years with a male predominance (58.2%). No patient had DSA prior to transplant. Cumulative dose of Thymoglobulin® was 4,26 ± 0,87 mg/kg, with an average duration of 5 ± 0,82 days. Lymphocyte depletion was maximal on the first day of infusion. Three patients had delayed graft function, at least one episode of bacterial infection in 56,4% of patients, 7 cases of CMV infections (12,7%) and 2 cases of CMV disease (3,6%). Graft survival rate was calculated for all patients with an average serum creatinine of 11,7 ± 3,6 mg/l during the last visit.

**Conclusion:**

although it is not indicated for first line treatment in patients with low immunological risk, Thymoglobulin® can nevertheless be prescribed at a lower dose, with similar efficacy and without exposure to a higher risk of rejection.

## Introduction

La transplantation rénale (TR) est le traitement de référence de l´insuffisance rénale chronique terminale nécessitant un traitement immunosuppresseur pour prévenir le rejet de l´allogreffon rénal. Le terme “traitement d´induction” se réfère au traitement immunosuppresseur prescrit spécifiquement dans la période per-opératoire, avec des effets qui se prolongent au-delà de la procédure de TR. Les recommandations internationales actuelles concernant le traitement d´induction en TR suggèrent l'utilisation d´agents biologiques tels que les anticorps monoclonaux et polyclonaux dirigés contre les lymphocytes T [1]. On distingue les globulines antithymocytaires (ATG® Sanofi Aventis, ou thymoglobuline®) et les antilymphoglobulines. La thymoglobuline®, est le médicament le plus couramment utilisé dans les schémas thérapeutiques d´induction en TR aux États-Unis [2], mais il n'existe à ce jour aucun schéma thérapeutique codifié par rapport au dosage, à la durée et au moment idéal pour initier le traitement. Bien que son efficacité ne soit pas contestée, ses nombreux effets secondaires à court et à long terme doivent faire évaluer le risque-bénéfice par rapport à d'autres thérapies moins toxiques. Le but de ce travail est d´étudier l´efficacité et la sureté de la thymoglobuline® en traitement d´induction chez des transplantés rénaux à faible risque immunologique.

## Méthodes

**Type et cadre de l´étude:** nous avons réalisé une étude rétrospective au sein du service de Néphrologie-Dialyse et Transplantation Rénale dans notre centre entre Janvier 2012 et Septembre 2017.

**Sélection des patients:** tous nos patients étaient à faible risque immunologique. Ce statut est défini par l´absence de TR antérieure avec absence d´anticorps dirigés contre le donneur (DSA). Ils ont tous reçu un traitement d´induction à base de thymoglobuline® et ont eu un suivi minimum de 12 mois.

**Conception de l´étude:** les paramètres étudiés pour chaque patient sont l´âge, le sexe, le poids, la taille, l´indice de masse corporelle (IMC), les comorbidités, la néphropathie initiale, le traitement d´épuration extra rénale antérieur et sa durée, le profil immunologique du receveur (phénomènes immunisants, nombre de mismatchs, la présence d´un DR en commun, la présence d´anticorps anti-HLA non DSA et le mismatch âge), le type de donneur, sa créatinine sérique, le statut CMV, le temps d´ischémie tiède, la dose cumulative de thymoglobuline® administrée, la dose journalière, la durée du traitement, la répartition des doses et le traitement d´entretien associé. Nous avons étudié également les paramètres biologiques (le taux de globules blancs (GB), de lymphocytes, de plaquettes, d´hémoglobine, de créatinine sérique et de protéinurie de 24h à J-1 puis chaque jour au cours de la première semaine de TR, à J-14, 1^er^ mois (M1), M3, M6, M9, M12 puis à chaque année de suivi), les complications post TR: les infections bactériennes, virales et fongiques, la reprise retardée de la fonction du greffon (RRF), les rejets cellulaires et humoraux aigus, les néoplasies et enfin la survie du greffon et du patient.

**Analyse statistique des données:** l´analyse statistique a été faite par le logiciel SPSS version 21.0. Les variables quantitatives ont été exprimées en moyennes et en écart-type et les variables qualitatives en effectif et pourcentage. Les données discrètes ont été décrites par leur fréquence exprimée en pourcentage avec son intervalle de confiance à 95% et ont été comparées par le test exact de Fisher vu le faible effectif. Les intervalles de confiance n´ont été réalisés qu´après transformation angulaire. Les données numériques ont été décrites parleur moyenne (avec son intervalle de confiance à 95% calculé par bootstrap) et l´écart-type. Les données continues ont été comparées par le test de Student après vérification de l´égalité des variances. Les analyses multivariées ont été réalisées par régression logistique.

## Résultats

Nous avons inclus 55 patients avec un suivi moyen de 38 ± 16 mois (12-77).

**Les caractéristiques cliniques et épidémiologiques**: l´âge moyen était de 39,1 ± 12,1 ans avec une prédominance masculine (58,2%). L´IMC était de 23,24 ± 4,14 kg/m^2^. La néphropathie initiale était indéterminée dans la moitié des cas (52,7%), d´origine glomérulaire dans 20% des cas et une polykystose rénale dans 7,3% des cas. L´hypertension artérielle (HTA), le diabète et l´obésité ont été notés respectivement dans 7,3%, 9,1% et 3,6% des cas. Avant la TR, la majorité des patients était en hémodialyse chronique (80%), 9,1% en dialyse péritonéale et 10,9% ont bénéficié d´une TR préemptive. La durée moyenne en dialyse était de 43,4 ± 42,7 mois (0-174).

**Le profil immunologique:** concernant les phénomènes immunisants avant la TR, 45,5% des patients ont bénéficié d´une transfusion sanguine et 56,5% des femmes ont eu au moins une grossesse. Aucun patient n´a bénéficié d´une greffe antérieure, par rapport à leur donneur respectif, 36,4% des patients sont semi-identiques alors que 12,7% sont identiques, 69,1 % des patients ont un DR en commun et 23,6% ont des anticorps antiHLA non DSA. Aucun patient n´avait de DSA avant la greffe. Par conséquent, tous nos patients ont été considérés comme étant à faible risque immunologique.

**Le profil du donneur:** la majorité des donneurs sont des donneurs vivants apparentés (81,8%), alors que ceux en état de mort encéphalique (EME) ne représentent que 18,2%. L´âge moyen des donneurs était de 38,4 ±13,6 ans. La créatinine sérique avant le don était de 9,8 ± 8,9 mg/l correspondant à un DFG estimé par MDRD à 93,2 ± 16,65 ml/min. Le mismatch âge était de 13,2 ± 9,1 ans. Le statut CMV a été étudié chez 49 patients, on note une majorité de couple D+/R+ (89,8%), les couples D+/R- et D-/R+ sont représentés à 4,1% alors qu´on note un seul couple D-/R-. Au cours de la procédure de TR, le temps moyen d´ischémie tiède était de 43 ± 10,5 min.

**Figure 1:**
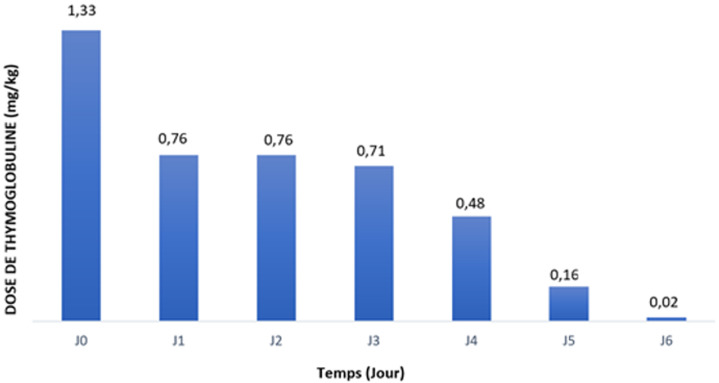
répartition des doses en fonction des jours (mg/kg)

**Traitement par les globulines antithymocytaires:** la molécule utilisée dans notre unité de TR est la thymoglobuline®. Le Tableau 1 représente les résultats concernant les doses de thymoglobuline® utilisées et la durée du traitement. La perfusion de thymoglobuline® a été bien tolérée sans réaction locale ou anaphylactique rapportée. La répartition des doses en fonction des jours est rapportée dans la Figure 1. Le traitement d´entretien était à base de mycophénolate mofétil (MMF) et de corticoïdes oraux chez tous nos patients. Ils ont été mis sous Tacrolimus et Ciclosporine dans respectivement 81,8% et 12,7% des cas, tandis que 3 patients HLA identiques n´ont pas reçu d´ anticalcineurines (ACN) (5,5%).

**Tableau 1:** caractéristiques du traitement par thymoglobuline^®^

Variables	Moyenne(M) ± Ecart type (ET)
Thymoglobuline^®^ dose cumulative (mg/kg)	4,26 ± 0,87
Thymoglobuline^®^ dose journalière (mg/kg/jr)	0,85 ± 0,18
Durée Traitement (jours)	5 ± 0,8

**Figure 2:**
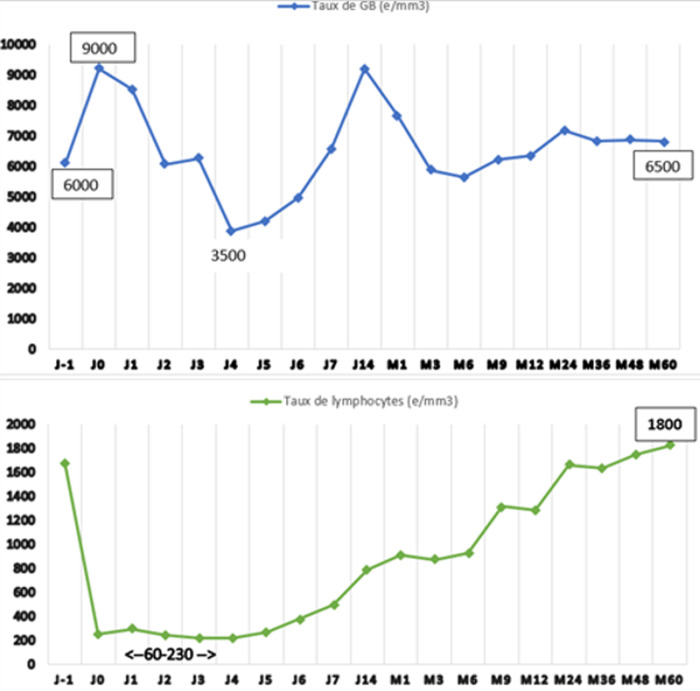
taux (e/mm^3^) de lymphocytes (courbe verte) et de globules blancs (courbe bleue) sur 5 ans

**Figure 3:**
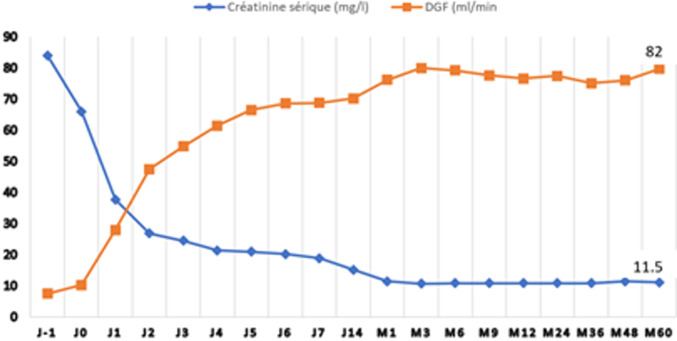
taux de créatinine sérique (mg/l) et DFG (ml/min)

**Suivi biologique:** on note une baisse progressive du taux de GB avec la moyenne la plus basse à J4 (3884,18 ± 1662,53 e/mm^3^), suivi d´une réascension progressive jusqu´à J14 pour atteindre 9185,63 ± 2928,23 e/mm^3^. La déplétion lymphocytaire est maximale au 1^er^ jour de perfusion avec une médiane à 130 [60-230] e/mm^3^ puis reste stable entre J0 et J5 (Figure 2). La thrombopénie s´installe à J2 du traitement, avec la moyenne la plus basse à J4 (130418 ± 44813 e/mm^3^). On note par la suite une augmentation progressive du taux pour atteindre une moyenne maximale à J14 (312836 ± 79255 e/mm^3^) avant de se stabiliser à partir du 3^e^ mois. Après une baisse initiale en postopératoire immédiat, on note une stabilisation du taux d´hémoglobine au cours de la 1^ère^ semaine de greffe. Notons également une amélioration du taux de créatinine sérique au cours du 1^er^ mois en post TR avec une moyenne à 11,5 ± 4,6 mg/l à M1. Le taux reste stable par la suite au cours de toute la période de suivi (Figure 3). Le taux de protéinurie se stabilise après 1 mois de la TR et la moyenne reste par la suite < 0,5 g/jour.

### Les complications

**La reprise retardée de la fonction du greffon:** nous avons recensé 3 cas de RRF dont 2 survenues chez des patients transplantés par un rein d´un donneur en EME. Le 3^e^ patient a présenté une RRF secondaire à une complication vasculaire en postopératoire (plicature de la veine rénale du greffon).

**Les complications infectieuses:** nous avons étudié les facteurs associés à la survenue de tout type d´infection, en analyse uni-variée, aucun facteur n´est ressorti statistiquement significatif (Tableau 2).

**Tableau 2:** facteurs associés à la survenue de tout type d´infection

Variables	Infection (n=43)	Absence d’infection (n=12)	OR	IC	p
**Age**	37,32±11,58	45,5±12,71	1,06	1-1,13	0,488
**Sexe (pourcentage)**			0,21	0,41-1,07	0,609
Homme	51,2	83,3			
Femme	48,8	16,7			
IMC (kg/m^2^)	22,7±3,8	25,1±4,6	1,16	0,98-1,37	0,81
Diabète n (pourcentage)	4 (9,3)	0 (0)			0,99
Durée en dialyse (mois)	43,25±42,7	43,91±44,6	1	0,98-1,01	0,96
Dose cumulative de thymoglobuline (mg/kg)	4,27±0,81	4,25±1,10	0,97	0,46-2,04	0,95
Dose journalière de thymoglobuline (mg/kg/jr)	0,86±0,18	0,81±0,18	0,24	0,07-8,08	0,43
Durée du traitement (jours)	5,02 ±0,83	5,16±0,83	1,24	0,56-2,74	0,59
Traitement d’entretien **(pourcentage)**			0,70	0,18-2,76	0,61
Tacrolimus	81,4	83,3			
Ciclosporine	11,6	16,7			
Pas d’ACN	7	0			

**Les infections bactériennes:** nous avons rapporté au moins un épisode d´infection bactérienne chez 56,4% de nos patients, avec un nombre moyen d´épisodes de 1,27 ± 1,7 (0-8). Le site infectieux était majoritairement urinaire (81%), suivi des infections pulmonaires et digestives dans respectivement 10% et 4,2% des cas. L´infection urinaire (IU) était récidivante chez 11 patients (35,4%). Le délai moyen de survenue du 1^er^ épisode infectieux était de 6,38 ± 12,72 mois (0,1-60). La dose totale de thymoglobuline (mg/kg) était légèrement plus élevée dans le groupe infection bactérienne (4,30 vs 4,22 avec p=0.75) (Tableau 3).

**Tableau 3:** facteurs associés à la survenue d´une infection bactérienne

Variables	Infection bactérienne (n=31)	Absence d’infection bactérienne (n=24)	OR	IC	p
**Age**	**36,61±12,51**	**42,33±11,22**	**1,04**	**0,99-1,09**	**0,088**
**Sexe (pourcentage)**			**0,27**	**0,86-0,87**	**0,029**
Homme	**54,8**	**75**			
Femme	**45,2**	**25**			
IMC (kg/m^2^)	**22,58±4,09**	**24,09±4,14**	**1,09**	**0,95-1,25**	**0,18**
Diabète**(pourcentage)**	**6,5**	**8,3**	**0,75**	**0,99-5,81**	**0,79**
Durée en dialyse (mois)	**45,09±46,42**	**41,20±38,28**	**0,99**	**0,98-1,01**	**0,73**
Dose cumulative de thymoglobuline (mk/kg)	**4,30±0,80**	**4,22±0,96**	**0,90**	**0,49-1,68**	**0,75**
Dose journalière de thymoglobuline (mg/kg/jr)	**0,87±0,20**	**0,83±0,15**	**0,32**	**0,01-6,18**	**0,45**
Durée du traitement (jours)	**5,06±0,89**	**5,04±0,75**	**0,96**	**0,50-1,85**	**0,91**
**Traitement d’entretien (pourcentage)**			**0,63**	**0,21-1,85**	**0,40**
Tacrolimus	**80,6**	**83,3**			
Ciclosporine	**9,7**	**16,7**			
Pas d’ACN	**9,7**	**0**			

**Les infections virales:** nous avons rapporté 7 cas d´infection à CMV (12,7%) et 2 cas de maladie à CMV (3,6%), ayant nécessité le recours au traitement curatif (Ganciclovir ou Valganciclovir). L´infection au virus BK (BKv) et à virus Herpès est retrouvée respectivement chez 25,5% et 20% des patients.

**Les infections fongiques:** deux patients (3,6%) ont présenté une infection fongique.

**Les complications immunologiques et néoplasiques:** nous avons recensé 1 cas de rejet humoral aigu et un autre cas de rejet cellulaire aigu, confirmés par les résultats histologiques à la ponction biopsie du greffon. Un seul patient a présenté une complication néoplasique à type de carcinome indifférencié du cavum après 44 mois de la TR.

**La survie:** la survie du greffon était notée chez tous nos patients après un délai de suivi moyen de 38 ± 16 mois, avec un taux de créatinine sérique moyen à 11,7 ± 3,6 mg/l au cours de la dernière consultation. Nous rapportons un seul décès dans notre cohorte, attribué à une cause cardiaque.

## Discussion

**Prescription de la thymoglobuline:** actuellement, les globulines anti-thymocytaires sont les traitements d'induction les plus utilisés dans le monde entier (60% des nouveaux transplantés rénaux aux Etats unis) [3]. Au cours des 30 dernières années, depuis sa première commercialisation, sa qualité, ses protocoles d'induction, les médicaments associés, ainsi que les caractéristiques des patients et les attentes des cliniciens ont considérablement évolués.

**La dose de thymoglobuline:** la déplétion des lymphocytes T ainsi que la survenue d´effets secondaires dépendent de la dose prescrite de thymoglobuline. Au fil du temps, cette dose a été réduite après la publication de résultats rassurants par rapport à une même efficacité à doses plus faibles tout en étant moins toxique [4]. Dans une étude publiée par Gaber *et al*. chez les receveurs à haut risque immunologique, une dose totale moyenne de 5,7 mg/kg a donné les mêmes résultats que chez les patients ayant reçu une moyenne de 10,3 mg/kg [4]. Dans notre étude, la dose journalière moyenne était de 0,85 mg/kg, donc plus basse que la dose administrée par Gaber *et al*.

Des protocoles récents publiés ont comparé de très faibles doses de thymoglobuline administrées à des patients à faible risque immunologique. Dans une petite série randomisée [5], deux schémas à faible dose ont été comparés (doses totales de 3,75 vs 2,25 mg/kg) chez des patients transplantés rénaux. Les deux régimes ont atteint un faible taux de rejet aigu prouvé par biopsie (17% contre 10%) avec potentiellement moins d'infections virales opportunistes chez le groupe à plus faible dose. Notre étude a donc montré encore une fois que les doses journalières beaucoup plus basse (0,85 mg/kg) peuvent avoir la même efficacité avec une dose cumulative comparable aux autres études (4.26 mg/kg).

Notre cohorte, considérée à faible risque immunologique, a reçu une dose cumulative moyenne de 4,26 ± 0,87 mg/kg avec des résultats satisfaisants (un taux de rejet aigu à 3,6% et une créatinine sérique moyenne à 3 ans à 10,9 mg/l), ce qui rejoint le travail de Laftavi *et al*. [6], qui ont étudié les avantages et les risques associés à la thymoglobuline à faible dose (3-5 mg/kg au total vs induction au basiliximab) dans une population à faible risque immunologique. Ils ont montré que chez les receveurs de reins par donneurs vivants, la survie du patient et du greffon à 8 ans n'était pas différente. Cependant, la thymoglobuline à faible dose était associée à un taux plus faible de RA (7,8 vs 35% basiliximab, p < 0,01) et une meilleure créatinine sérique à 3 et 5 ans (12 vs 15, p = 0,02 et 11,8 vs 15,4, p = 0,04, respectivement). Pour les transplantés rénaux par un rein de cadavre, la thymoglobuline à faible dose était associée à une meilleure survie du greffon à long terme (86 vs 76% avec le basiliximab, p =0,02).

La dose efficace la plus faible est toujours sujet de débat. Un aperçu utile a été donné par un groupe hollandais qui a évalué l'effet des différentes doses de thymoglobuline sur les cellules T, les cellules B et les cellules NK [7]. A 1,5 mg/kg de dose totale, la déplétion des cellules T et cellules NK se prolongeait une semaine après la transplantation, mais un mois plus tard, leur taux était revenu à son niveau de base. Les patients recevant 3 mg/kg avaient encore un taux bas de cellules T après un mois, revenu aux valeurs de base après un an. La lymphopénie des cellules T se prolonge au cours de la première année post-transplantation chez ceux qui ont reçu une dose totale de 6 mg/kg. Nos résultats sont similaires à ceux de l´étude hollandaise avec une déplétion lymphocytaire de même niveau à court terme.

**Durée optimale du traitement:** dans notre étude, la durée d´administration de la thymoglobuline était de 5 ± 0,8 jours. Elle a diminué au fil du temps et dure généralement 3 à 5 jours [8]. L´objectif étant de raccourcir la durée d´hospitalisation.

**Début du traitement:** dans notre unité, l´initiation du traitement par la thymoglobuline se fait au moment de l´anesthésie, 2 heures avant le déclampage de l´artère du greffon. Goggins *et al*. ont rapporté que ce timing peut minimiser le risque de RRF [9] en agissant sur les lésions d´ischémie/reperfusion (IRI). Il n´existe à ce jour pas de consensus de sorte que l'administration postopératoire peut également être appropriée.

**Administration intermittente ou quotidienne:** nous avons prescrit une dose initiale de 1,25 mg/kg le jour de la TR, les doses ultérieures sont conditionnées par le taux total de lymphocytes qui est réévalué chaque jour au cours de la première semaine de greffe. Deux études monocentriques ont montré que la prescription intermittente de thymoglobuline en se basant sur le taux de lymphocytes T CD3+ a abouti à une efficacité similaire par rapport à une prescription quotidienne fixe, tout en réduisant la dose totale de thymoglobuline [10,11] ainsi que son coût [11].

**Site d'infusion:** il est recommandé d´administrer la thymoglobuline® en perfusion intraveineuse via une veine à haut débit (veine centrale ou fistule artérioveineuse) pendant au moins six heures pour la première perfusion et au moins quatre heures pour les doses suivantes [12] afin de réduire le risque de réactions associées à la perfusion. Au niveau de notre unité, la thymoglobuline® est administrée par voie veineuse centrale, posée au bloc opératoire après l´anesthésie générale.

**Prévention du rejet aigu:** l´utilisation de l'induction par thymoglobuline dans des schémas modernes ont été associé à une réduction significative du taux de RA [13,14]. Dans notre série, nous avons relevé 2 épisodes de rejet, dont un chez une patiente identique traitée par thymoglobuline en induction sans ACN en traitement d´entretien. Chez les patients à faible risque immunologique [15,16], le taux de RA et de survie du patient et du greffon semble similaires avec les IL-2RA ou la thymoglobuline quel que soit le traitement d'entretien, mais avec une meilleure tolérance pour l'induction avec l'IL-2RA [17,18]. Récemment, une étude prospective randomisée a comparé l´induction par la thymoglobuline, par l´IL-2RA (basiliximab) et par l´Alemtuzumab (Campath) chez des patients transplantés rénaux recevant du tacrolimus avec le MMF et un cycle de cinq jours de corticostéroïdes [19]. La supériorité d'alemtuzumab par rapport au taux de RA précoce confirmé par biopsie était limitée aux patients présentant un faible risque immunologique. Chez les patients à haut risque, l´Alemtuzumab et la thymoglobuline ont démontré une efficacité similaire et un taux similaire d'événements indésirables [19].

**Thymoglobuline et RRF:** la thymoglobuline offre le potentiel d'améliorer l'IRI en inhibant l´activation et l´adhésion de cellules inflammatoires et les médiateurs via la déplétion en leucocytes et en lymphocytes T [19]. Nous avons recensé 3 cas (5,4%) de RRF avec un recours à la dialyse au cours de la première semaine post greffe, dont une RRF chez un patient ayant eu une plicature de la veine rénale du greffon avec reprise chirurgicale. Goggins *et al*. [9] ont signalé que l´administration peropératoire de thymoglobuline avant la reperfusion réduit significativement l'incidence de la RRF par rapport à l´administration post-opératoire (3,5%, vs 14,8%, p <0,05), ainsi qu´une amélioration de la fonction rénale aux jours 10 et 14 post-transplantation avec une hospitalisation plus courte. En outre, une approche intéressante chez les patients à risque de RRF peut être d´utiliser l'induction par thymoglobuline pour retarder l'introduction des ACN et ainsi augmenter les chances d´une reprise immédiate de la fonction du greffon. Dans notre centre, l´introduction des ACN se fait généralement 2 à 3 jours après la TR.

**Protocole sans corticostéroïdes:** le protocole de notre service consiste à administrer une corticothérapie les 3 premiers jours de la TR sous forme de bolus quotidien (Méthylprednisolone 500 mg à J1, 120 mg à J2 et 120 mg à J3) relayé par une corticothérapie orale à 20 mg/jour avec une dégression progressive sur 8 mois jusqu´à atteindre 5 mg/jour, que le patient garde à vie. L´utilisation de la thymoglobuline pour faciliter le retrait des corticoïdes a été étudiée. Dans une grande étude randomisée en double aveugle, l'incidence du RA a été évaluée chez 500 patients recevant une trithérapie avec de la ciclosporine, du MMF et un protocole standard ou à moitié dose de corticoïdes avec arrêt à 3 mois [20]. Parmi une sous-population de 104 patients ayant reçu l´induction par la thymoglobuline, l´incidence du RA était similaire quel que soit le protocole de corticothérapie (13,4% dans le groupe à faible dose vs 11,5% dans le groupe protocole standard) [21]. Ces résultats suggèrent que la réduction de la dose de corticoïdes est possible sans taux de RA excessif chez les patients ayant reçu un traitement d'induction à base de thymoglobuline [21].

**Surveillance et sécurité:** en l'absence d'un schéma standard d´administration de la thymoglobuline, ses effets hématologiques et immunologiques doivent être monitorés à court et à long terme. À court terme, la surveillance de la déplétion des lymphocytes T oriente l'ajustement de la dose quotidienne. La déplétion en lymphocytes T peut être évaluée soit par la numération des globules blancs ou par cytométrie en flux avec analyse des sous-populations de cellules T. Le taux de CD3+, effectué quotidiennement ou trois fois par semaine, est le test le plus couramment utilisé. Le but est de maintenir le nombre de lymphocytes inférieur à 200 e/mm^3^ et/ou le taux de CD3+ inférieur à 20 e/mm^3^. Il est généralement admis que ce niveau de déplétion des cellules T est associé à l'absence de rejet cellulaire aigu lors de la phase d´induction [12].

**Effets secondaires:** la perfusion de la thymoglobuline est généralement bien tolérée, sans anaphylaxie ou réactions locales au site de perfusion. Cependant, les réactions telles qu´une fièvre, une éruption cutanée, une leucopénie et une thrombocytopénie sont observés chez 25% des patients [22]. Nous n´avons rapporté aucune réaction anaphylactique dans notre série. Les événements hématologiques tels que la thrombocytopénie (<50000 e/L) se produit à une fréquence similaire et se résout généralement sans interruption du traitement [22]. Dans notre série, la thrombopénie s´installe à J2 du traitement, avec la moyenne la plus basse à J4 (130 418 e/mm^3^). On note par la suite une augmentation progressive du taux de plaquettes pour atteindre une moyenne maximale à J14 (312 836 e/mm^3^) avant de se stabiliser à partir du 3^e^ mois. Une thrombopénie sévère à 36 000 e/mm^3^ a été notée chez un seul patient à J5 sans évènement hémorragique, ayant nécessité l´arrêt du traitement après 2 doses. La maladie sérique avec fièvre, arthralgies, adénopathie et avec ou sans éruption cutanée et survenant 7 à 11 jours après la TR, est maintenant moins fréquente (0,25%) en raison de la réduction des doses et des durées d'exposition [23]. La déplétion des cellules T induite par la thymoglobuline, qui est le signe de l´efficacité du traitement, est suivie par une phase de reconstitution immunitaire, avec à la fois une émigration thymique de nouveaux lymphocytes T naïfs et une prolifération de cellules T mémoires résistants à la déplétion [24]. Cette phase de reconstitution immunitaire se produit lentement et peut être prolongée pour plusieurs années. Dans notre série, le taux de lymphocytes a augmenté progressivement après la 1^ere^ semaine, ce n´est qu´après 2 ans que le taux devient > 1500 e/mm^3^. Les facteurs de risques d´un déficit de reconstitution ne sont pas bien définis, seul l´âge avancé est rapporté [25].

**Infections bactériennes:** l'impact de la thymoglobuline sur les infections bactériennes n'est pas clair. Plusieurs cofacteurs sont généralement présents, y compris les complications techniques de la chirurgie, urinaires et vasculaires, les cathéters et l´association à d´autres thérapies immunosuppressives. Cependant, les infections bactériennes sont la forme la plus courante des infections rapportées après le traitement d'induction par thymoglobuline [26,27], elle a été notée chez 56,4% de nos patients avec en 1^er^ lieu les infections urinaires (81%), ce qui rejoint les données de la littérature suivies par les infections des plaies [28,29]. Les bactériémies et les pneumonies ont également été rapportées [30]. Les enterobactéries (Escherichia coli et Enterococcus) sont les espèces uropathogènes les plus fréquemment isolées [26]. Nous n´avons pas retrouvé d´association significative entre la dose totale de thymoglobuline et la survenue d´une infection bactérienne. Dans la plupart des études prospectives, la thymoglobuline n´a pas été associée à un risque accru d´infection bactérienne, par rapport à un protocole sans induction ou par rapport à d'autres traitements d'induction (l'IL-2RA et alemtuzumab) [31-33].

**Infections virales:** l'association entre les globulines anti thymocytaires et les infections virales est bien établie. Après traitement par la thymoglobuline, une augmentation de l´incidence et de la gravité de l'infection à CMV [13,15] a été signalée en particulier chez les patients n´ayant pas reçu de traitement prophylactique. Tous nos patients ont reçu un traitement prophylactique anti CMV pendant 6 mois (Valaciclovir ou Valganciclovir) indépendamment de leur statut D/R. Nous avons rapporté 7 cas d´infection à CMV (12,7%) et 2 cas de maladie à CMV (3,6%), ayant nécessité le recours au traitement curatif (Ganciclovir ou Valganciclovir). En effet, dans la plupart des études prospectives randomisées, en comparant la thymoglobuline avec d'autres agents d'induction, l'incidence de l´infection à CMV n´est pas plus élevée lorsque le traitement d´induction était associé à une prophylaxie adéquate contre le CMV [34,35].

Une étude prospective [36] a suggéré un lien entre la thymoglobuline et l´infection à BKv. Ceci peut être particulièrement fréquent chez les patients recevant une dose plus élevée de thymoglobuline (> 500 mg) [37]. Le dépistage des infections à BKv pendant au moins six mois après la transplantation rénale est recommandé. L´effet des fortes doses de thymoglobuline s´explique par le fait que la réplication et la néphropathie à BKv sont associées à une augmentation de l´immunosuppression [36]. Effectivement, tous les patients ayant réactivé ou présenté une néphropathie à BKv dans notre série (25,5%) ont bien répondu après allégement de l´immunosuppression.

**Risque néoplasique:** la conséquence de la lymphopénie T CD4+ sur le risque néoplasique est toujours controversée, et semble être associé à une incidence élevée pour certains types de néoplasies [38] mais pas d´autres [39]. Une série d'analyses de registre a montré que le traitement par globulines antithymocytaires est associé à une incidence plus élevée de maladie lymphoproliférative post-transplantation [40].

**Limites de l´étude:** la nature rétrospective de notre étude représente une de ses principales faiblesses puisqu´elle la rend propice à l´omission de beaucoup de données qu´on a cherchées dans les anciens dossiers des patients. Une autre limite est représentée par la petite taille de la population, ce qui peut avoir comme effet de réduire la puissance de l´étude. Finalement, le suivi à long terme manque dans cette étude, ce qui fait partie de ses limites.

## Conclusion

Grâce à ses mécanismes d'action uniques, la thymoglobuline® reste un acteur majeur dans l´arsenal thérapeutique immunosuppresseur en TR, surtout chez les patients à haut risque immunologique. Bien qu´elle ne soit pas indiquée en 1^ère^ intention chez les patients à faible risque immunologique, elle peut néanmoins être prescrite à dose plus faible, avec une efficacité similaire et sans exposition à un risque de rejet plus élevé. Les complications d´ordre infectieux peuvent être limitées grâce aux traitements prophylactiques et un monitorage strict en post TR. Notre étude confirme l´efficacité et le bon profil de sécurité de la thymoglobuline® en traitement d´induction en transplantation rénale.

### Etat des connaissances sur le sujet

- La thymoglobuline® est un anticorps polyclonal indiqué en traitement d´induction en transplantation rénale;
- Son efficacité non contestée, ses nombreux effets secondaires à court et à long terme doivent faire évaluer le risque-bénéfice par rapport à d'autres thérapies moins toxiques.

### Contribution de notre étude à la connaissance

- La thymoglobuline®, en traitement d´induction chez des transplantés rénaux à faible risque immunologique, peut être administrée à des doses journalières très faibles sans perdre son efficacité;
- La thymoglobuline® n´a pas été associée à un risque accru d´infection bactérienne;
- Le lien entre la thymoglobuline® et l´infection à BK virus est rapporté ainsi qu´une incidence plus élevée de maladie lymphoproliférative post-transplantation.
